# Concomitant Use of Selective Serotonin Reuptake Inhibitors With Oral Anticoagulants and Risk of Major Bleeding

**DOI:** 10.1001/jamanetworkopen.2024.3208

**Published:** 2024-03-22

**Authors:** Alvi A. Rahman, Robert W. Platt, Sarah Beradid, Jean-François Boivin, Soham Rej, Christel Renoux

**Affiliations:** 1 Department of Epidemiology, Biostatistics and Occupational Health, McGill University, Montreal, Quebec, Canada; 2 Centre for Clinical Epidemiology, Lady Davis Institute for Medical Research, Jewish General Hospital, Montreal, Quebec, Canada; 3 Department of Pediatrics, McGill University, Montreal, Quebec, Canada; 4 Department of Psychiatry, McGill University, Montreal, Quebec, Canada; 5 Department of Neurology and Neurosurgery, McGill University, Montreal, Quebec, Canada; 6 Department of Medicine, McGill University, Montreal, Quebec, Canada

## Abstract

**Importance:**

Selective serotonin reuptake inhibitors (SSRIs) are commonly prescribed antidepressants associated with a small increased risk of major bleeding. However, the risk of bleeding associated with the concomitant use of SSRIs and oral anticoagulants (OACs) has not been well characterized.

**Objectives:**

To assess whether concomitant use of SSRIs with OACs is associated with an increased risk of major bleeding compared with OAC use alone, describe how the risk varies with duration of use, and identify key clinical characteristics modifying this risk.

**Design, Setting, and Participants:**

A population-based, nested case-control study was conducted among patients with atrial fibrillation initiating OACs between January 2, 1998, and March 29, 2021. Patients were from approximately 2000 general practices in the UK contributing to the Clinical Practice Research Datalink. With the use of risk-set sampling, for each case of major bleeding during follow-up, up to 30 controls were selected from risk sets defined by the case and matched on age, sex, cohort entry date, and follow-up duration.

**Exposures:**

Concomitant use of SSRIs and OACs (direct OACs and vitamin K antagonists [VKAs]) compared with OAC use alone.

**Main Outcomes and Measures:**

The main outcome was incidence rate ratios (IRRs) of hospitalization for bleeding or death due to bleeding.

**Results:**

There were 42 190 patients with major bleeding (mean [SD] age, 74.2 [9.3] years; 59.8% men) matched to 1 156 641 controls (mean [SD] age, 74.2 [9.3] years; 59.8% men). Concomitant use of SSRIs and OACs was associated with an increased risk of major bleeding compared with OACs alone (IRR, 1.33; 95% CI, 1.24-1.42). The risk peaked during the initial months of treatment (first 30 days of use: IRR, 1.74; 95% CI, 1.37-2.22) and persisted for up to 6 months. The risk did not vary with age, sex, history of bleeding, chronic kidney disease, and potency of SSRIs. An association was present both with concomitant use of SSRIs and direct OACs compared with direct OAC use alone (IRR, 1.25; 95% CI, 1.12-1.40) and concomitant use of SSRIs and VKAs compared with VKA use alone (IRR, 1.36; 95% CI, 1.25-1.47).

**Conclusions and Relevance:**

This study suggests that among patients with atrial fibrillation, concomitant use of SSRIs and OACs was associated with an increased risk of major bleeding compared with OAC use alone, requiring close monitoring and management of risk factors for bleeding, particularly in the first few months of use.

## Key Points

**Question:**

Is there an association between concomitant use of selective serotonin reuptake inhibitors (SSRIs) and oral anticoagulants (OACs) and the risk of major bleeding among patients with atrial fibrillation compared with OAC use alone?

**Findings:**

In this nested case-control study comprising 42 190 cases with major bleeding matched to 1 156 641 controls, concomitant SSRI and OAC use was associated with a 33% increased risk of major bleeding compared with OAC use alone; this risk was highest in the first few months of concomitant use and was substantially lower after 6 months.

**Meaning:**

This study suggests that concomitant use of SSRIs and OACs may be a risk factor for bleeding and should be closely monitored, particularly within the initial months of treatment.

## Teaser

This case-control study assesses whether the concomitant use of selective serotonin reuptake inhibitors (SSRIs) and oral anticoagulants was associated with increased risk of major bleeding vs oral anticoagulant use alone among UK adults.

## Introduction

Antidepressant medications are among the most frequently prescribed class of drugs worldwide, with up to 19% of individuals aged 60 years or older in the US reporting use of an antidepressant over the past 30 days.^1^ Selective serotonin reuptake inhibitors (SSRIs) are the most widely used antidepressant medications and are often recommended over other classes of antidepressants for the treatment of major depressive disorder due to their comparable efficacy and favorable safety profile.^2,3^ However, SSRIs have been shown to increase the risk of major bleeding,^4,5,6,7,8,9,10,11,12,13,14^ possibly owing to their inhibition of platelet activation during hemostasis.^2^ Although the absolute risk remains low for most individuals who use SSRIs,^11,12,15^ coprescription with drugs such as oral anticoagulants (OACs) may be consequential. Concomitant use of SSRIs and OACs is common given the prevalence of mental health disorders.^16^

Some observational studies have assessed the association between concomitant use of SSRIs and OACs and the risk of major bleeding. However, some had notable limitations, including exposure misclassification,^17^ possible informative censoring,^18,19^ residual confounding,^19,20,21^ and limited statistical power.^20,22,23,24^ Gaps in evidence that may inform the coprescription of SSRIs and OACs include whether the risk varies with demographic or clinical characteristics or between direct OACs (DOACs) and vitamin K antagonists (VKAs). In addition, data on the risk of specific types of major bleeding are limited.^25^

To address these knowledge gaps, we conducted a population-based, nested case-control study to assess whether the concomitant use of SSRIs and OACs was associated with the risk of major bleeding compared with OAC use alone among patients with atrial fibrillation (AF). We also assessed whether the risk varied by duration of use, relevant demographic and other risk factors, potency of SSRIs, and OAC type.

## Methods

### Data Source

In this population-based, nested case-control study, we used the UK Clinical Practice Research Datalink (CPRD GOLD and Aurum databases), a large primary care database of electronic medical records that contains demographic and lifestyle information, medical diagnoses, prescriptions, and referrals for more than 60 million patients from more than 2000 general practices.^26,27^ These data are representative of the UK population in terms of age, sex, and race and ethnicity.^26,27^ Drug prescriptions issued by the general practitioner are automatically recorded at the time of prescription.^26,27^ Quality control audits of the CPRD are regularly conducted to ensure the accuracy and completeness of data.^26,27^ The CPRD was linked with the Hospital Episodes Statistics repository, which contains details of inpatient and day case admissions,^28^ and the Office for National Statistics database, which contains electronic death certificates.^29^ The study protocol was approved by the CPRD Research Data Governance (No. 22_001906) and the Research Ethics Board of the Jewish General Hospital in Montreal, Canada, which also waived the need for patient informed consent as the data were deidentified. The study followed the Strengthening the Reporting of Observational Studies in Epidemiology (STROBE) reporting guideline.^30^

### Study Design and Population

We conducted a population-based study with a nested case-control approach to analysis because of its computational efficiency compared with a full-cohort analysis given the time-varying nature of both medications of interest, the size of the cohort, and the long duration of follow-up.^31^ We first identified all patients aged 18 years or older with an incident diagnosis of AF between January 2, 1998, and March 29, 2021, and at least 1 year of registration with the practice before AF diagnosis. From this base cohort, we selected those with a prescription for an OAC (apixaban, dabigatran, edoxaban, rivaroxaban, or warfarin) after AF diagnosis, with the date of first prescription defined as study cohort entry. We excluded patients who received OACs any time before cohort entry or SSRIs 6 months prior to cohort entry. We also excluded patients with hyperthyroidism in the year prior to cohort entry because AF in association with hyperthyroidism rarely requires long-term oral anticoagulation. Patients meeting these criteria were followed up until a first major bleeding event, death, end of registration with the practice, or end of the study period (March 29, 2021), whichever occurred first.

### Selection of Cases and Controls

We identified cases as patients with a first recorded diagnosis of major bleeding during follow-up, defined as hospitalization with a primary diagnosis of major bleeding or death with bleeding as the primary cause, using relevant *International Statistical Classification of Diseases and Related Health Problems, Tenth Revision* (*ICD-10*) codes (eTable 1 in Supplement 1); elective hospitalizations were not considered. *ICD-10* codes for bleeding have shown good positive predictive values between 81% and 95%.^32,33,34^ The index date was the date of hospital admission. For each case, we randomly selected up to 30 controls among the cohort members from the risk sets defined by the case. Each risk set, at each case’s index date, included all individuals who did not experience major bleeding and thus were still at risk up to that point in follow-up time, matched on age, sex, calendar date of cohort entry (±6 months), and duration of follow-up. Thus, as per the risk-set sampling approach, cases were eligible for selection as controls prior to becoming a case, and patients may have been selected as controls for multiple cases.^35,36^ The index date for controls was the date resulting in the same duration of follow-up for cases and controls.

### Exposure Definition

We identified prescriptions of SSRIs (citalopram, escitalopram, fluoxetine, fluvoxamine, paroxetine, or sertraline) and OACs for all cases and their matched controls between cohort entry and the index date. Exposure was defined in 4 mutually exclusive categories: concomitant use of SSRIs and OACs, OAC use alone, nonuse, and other use. We considered patients as concomitant users of SSRIs and OACs if the duration of their last prescription for both medications covered or ended 30 days before the index date. Similarly, we considered patients as users of OACs alone if their last prescription for an OAC covered or ended 30 days before the index date, without a prescription for an SSRI in this period—this was the reference category. Users of SSRIs alone, non-SSRI antidepressants alone, or non-SSRI antidepressants concomitantly with SSRIs and/or OACs were classified separately (other use). Finally, nonusers were those not exposed to any medications of interest on or 30 days before the index date.

### Covariates

We adjusted all models for the following comorbidities based on substantive knowledge, measured at or earlier than 365 days (1 year) before the index date: smoking, alcohol abuse, body mass index (BMI; calculated as weight in kilograms divided by height in meters squared) (<25, 25-29.9, or ≥30.0), depression, hypertension, diabetes, stroke or transient ischemic attack, coronary artery disease, congestive heart failure, peripheral arterial disease, disorders of hemostasis, cancer (other than nonmelanoma skin cancer), liver disease, chronic kidney disease, chronic obstructive pulmonary disease, anemia, and venous thromboembolism. We also included history of bleeding at any time before cohort entry and the time between incident AF diagnosis and first OAC prescription. Diabetes and hypertension were defined using diagnostic codes or relevant medications. All models were also adjusted for use of the following drugs measured between 365 days (1 year) and 730 days (2 years) prior to the index date: angiotensin-converting enzyme inhibitors, angiotensin II receptor blockers, β-blockers, calcium channel blockers, thiazide diuretics, other diuretics, antiplatelets, lipid-lowering drugs (including statins), antipsychotics, non-SSRI antidepressants, nonsteroidal anti-inflammatory drugs, proton pump inhibitors, and H_2_ receptor blockers. We considered the number of hospitalizations in the year before cohort entry as a surrogate marker for overall health. Finally, we adjusted for socioeconomic status using the Index of Multiple Deprivation, categorized in deciles.^37^

### Statistical Analysis

We used conditional logistic regression to compute odds ratios of major bleeding associated with concomitant use of SSRIs and OACs compared with OAC use alone, adjusting for the covariates listed. In a nested case-control approach, odds ratios are unbiased estimators of incidence rate ratios (IRRs) with very limited loss in precision.^36^ In secondary analyses, we assessed whether the risk of major bleeding varied according to age, sex, chronic kidney disease, history of bleeding, type of OAC (DOACs or VKAs), and potency of SSRIs (strong or moderate serotonin reuptake inhibitors based on the dissociation constant).^38^ Next, among patients continuously exposed to OACs and concomitantly exposed to SSRIs and OACs at the index date, we investigated whether the risk of major bleeding varied with the duration of continuous concomitant use of SSRIs in 3 prespecified categories (≤30 days, 31-180 days, or >180 days) compared with OAC use alone. These categories were selected because SSRIs were reported to exert antiplatelet action as early as 2 to 3 weeks after initiation.^39,40^ We defined continuous exposure to SSRIs and OACs separately, allowing a 30-day grace period between consecutive prescriptions where patients were still considered exposed. Patients were then considered concomitant users on any given day if exposed to both drugs on that day. In addition, we used a restricted cubic spline with 5 interior knots to produce a smooth curve of the IRR as a function of continuous duration of use. We also estimated the risk in specific anatomical locations, including gastrointestinal bleeding, intracranial hemorrhage, and other major bleeding. We assessed the risk of any bleeding associated with concomitant use of SSRIs and OACs. For this analysis, we repeated the selection of cases and controls already described, with cases defined using relevant diagnostic codes in primary electronic medical records. Finally, we assessed whether an interaction was present between SSRIs and OACs with respect to the risk of major bleeding on both the additive and multiplicative scales (eMethods 1 in Supplement 1). In other words, we assessed whether the joint association of the 2 exposures departed from the sum or product of their individual associations with the risk of bleeding, although an additive interaction has been described as most indicative of biological or mechanistic interaction.^41,42^

We performed 4 sensitivity analyses to assess the robustness of the results. First, to explore potential exposure misclassification, we considered only prescriptions that covered the index date and, next, those that covered or ended within 15 days before the index date. Second, to account for the potential adjustment for covariates affected by exposure, all covariates were measured at or prior to cohort entry. Third, we implemented multiple imputation by chained equations for missing values of BMI and smoking, combining results from 5 imputed datasets.^43^ Fourth, we repeated the analysis by type of OAC, excluding patients with a history of valvular surgery or rheumatic valvular disease before cohort entry because DOACs are not indicated for patients with valvular AF.^44,45,46^

We conducted a supplementary time-conditional propensity score–matched analysis to further explore the potential for residual confounding.^47,48^ In brief, among the base cohort of patients with incident AF initiating OACs, we matched each patient initiating SSRIs to a patient using OACs alone up to that point in time with the same age (±1 year), sex, calendar date of OAC initiation (±1 year), and time-conditional propensity score (eMethods 2 in Supplement 1). Finally, we conducted a post hoc analysis, repeating the primary analysis with additional adjustment for the following comedications reported to interact with OACs, measured between 1 and 2 years before the index date: clarithromycin, erythromycin, penicillin, azole antifungals, quinidine, amiodarone, dronedarone, propafenone, allopurinol, oral corticosteroids, tamoxifen, valproic acid, cyclosporin, tacrolimus, disulfiram, methylphenidate, and sulfamethoxazole. All analyses were performed with a 2-sided hypothesis test, and *P* < .05 was considered statistically significant, without adjustment for multiple comparisons, using SAS, version 9.4 (SAS Institute Inc).

## Results

After applying all eligibility criteria, the cohort included 331 305 patients (mean [SD] age, 73.7 [10.8] years; 57.1% men) with incident AF initiating OACs (eFigure in Supplement 1). During a mean (SD) follow-up of 4.6 (4.0) years, 42 391 patients were hospitalized with major bleeding, yielding an incidence rate of 27.9 per 1000 person-years (95% CI, 27.7-28.2 per 1000 person-years). Among those, 42 190 cases (mean [SD] age, 74.2 [9.3] years; 59.8% men) were matched to 1 156 641 controls (mean [SD] age, 74.2 [9.3] years; 59.8% men). As anticipated, risk factors for major bleeding were more prevalent among cases than controls (Table 1).

**Table 1.** Characteristics of Cases With Major Bleeding and Matched Controls^a^

Characteristic	Participants, No. (%)	
	Cases (n = 42 190)	Controls (n = 1 156 641)
Age, mean (SD), y	74.2 (9.3)	74.2 (9.3)
18-49	555 (1.3)	7066 (1.3)
50-59	2397 (5.7)	57 855 (5.7)
60-69	8713 (20.7)	239 196 (20.7)
70-79	17 599 (41.7)	501 022 (41.7)
≥80	12 926 (30.6)	351 502 (30.6)
Sex		
Female	16 979 (40.2)	459 262 (40.2)
Male	25 211 (59.8)	697 379 (59.8)
Year of cohort entry		
1998-2004	9525 (22.6)	232 013 (22.6)
2005-2008	8976 (21.3)	245 790 (21.3)
2009-2012	9065 (21.5)	255 651 (21.5)
2013-2016	9894 (23.5)	285 678 (23.5)
2017-2022	4730 (11.2)	137 509 (11.2)
Time to initiation of OAC, d		
≤30	18 766 (44.5)	539 029 (46.6)
31-120	10 808 (25.6)	300 987 (26.0)
>120	12 616 (29.9)	316 625 (27.4)
Comorbidities and risk factors		
BMI		
<25	11 168 (26.5)	293 526 (25.4)
25-29	14 391 (34.1)	411 354 (35.6)
≥30	12 394 (29.4)	330 043 (28.5)
Unknown	4237 (10.0)	121 718 (10.5)
Smoking		
Never	18 631 (44.2)	526 064 (45.5)
Ever	22 313 (52.9)	593 534 (51.3)
Unknown	1246 (3.0)	37 043 (3.2)
Alcohol abuse	2719 (6.4)	66 458 (5.7)
Hypertension	34 521 (81.8)	913 437 (79.0)
Coronary artery disease	13 259 (31.4)	327 272 (28.3)
Congestive heart failure	9158 (21.7)	219 816 (19.0)
Peripheral arterial disease	2759 (6.5)	65 926 (5.7)
Venous thromboembolism	2769 (6.6)	65 374 (5.7)
Stroke or TIA	8010 (19.0)	199 369 (17.2)
Diabetes	10 832 (25.7)	274 659 (23.7)
History of bleeding^b^	6038 (14.3)	106 023 (9.2)
Anemia	7324 (17.4)	157 825 (13.6)
Disorders of hemostasis	520 (1.2)	10 818 (0.9)
Cancer (other than nonmelanoma skin cancer)	8269 (19.6)	184 599 (16.0)
Depression	7117 (16.9)	169 590 (14.7)
Chronic obstructive pulmonary disease	5267 (12.5)	125 647 (10.9)
Liver disease	1325 (3.1)	28 896 (2.5)
Chronic kidney disease	12 626 (29.9)	312 373 (27.0)
Medications		
ACEIs	17 977 (42.6)	481 133 (41.6)
ARBs	7879 (18.7)	205 098 (17.7)
β-Blockers	22 663 (53.7)	619 755 (53.6)
CCBs	14 908 (35.3)	394 916 (34.1)
Thiazide diuretics	8119 (19.2)	225 431 (19.5)
Other diuretics	15 792 (37.4)	373 429 (32.3)
Antiplatelet agents	13 557 (32.1)	357 762 (30.9)
Statins or LLDs	23 573 (55.9)	635 145 (54.9)
Non-SSRI antidepressants	3913 (9.3)	90 857 (7.9)
Antipsychotic drugs	1915 (4.5)	45 191 (3.9)
NSAIDs	3843 (9.1)	104 600 (9.0)
Proton pump inhibitors	15 192 (36.0)	387 797 (33.5)
H_2_ receptor blockers	2251 (5.3)	49 444 (4.3)
No. of hospitalizations^c^		
0	14 293 (33.9)	446 231 (38.6)
1	14 303 (33.9)	399 022 (34.5)
≥2	13 594 (32.2)	311 388 (26.9)
Index of multiple deprivation, deciles		
1	3743 (8.9)	106 126 (9.2)
2	4266 (10.1)	115 166 (10.0)
3	3610 (8.6)	98 691 (8.5)
4	4155 (9.8)	116 509 (10.1)
5	4249 (10.1)	119 044 (10.3)
6	4595 (10.9)	131 405 (11.4)
7	4490 (10.6)	122 857 (10.6)
8	4096 (9.7)	110 226 (9.5)
9	4484 (10.6)	119 159 (10.3)
10	4502 (10.7)	117 458 (10.2)

Abbreviations: ACEIs, angiotensin-converting enzyme inhibitors; ARBs, angiotensin II receptor blockers; BMI, body mass index (calculated as weight in kilograms divided by height in meters squared); CCBs, calcium channel blockers; LLDs, lipid-lowering drugs; NSAIDs, nonsteroidal anti-inflammatory drugs; OAC, oral anticoagulant; SSRI, selective serotonin reuptake inhibitor; TIA, transient ischemic attack.

^a^ Cases and controls were matched on age, sex, and calendar year of cohort entry. For controls, mean values and percentages are weighted by the inverse of the number of controls matched to each case.

^b^ Measured any time prior to cohort entry.

^c^ Measured in the year prior to cohort entry.

**Figure 1.**
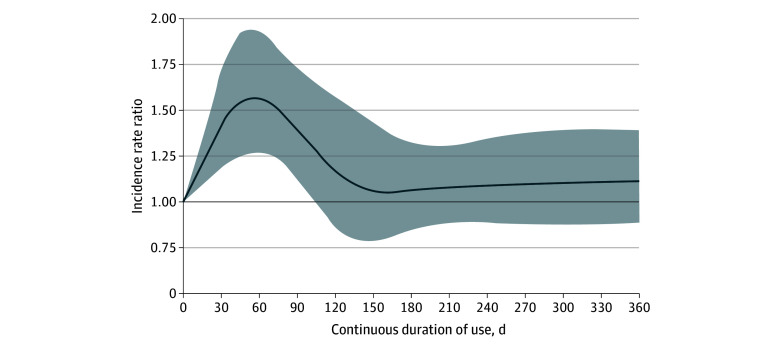
Restricted Cubic Spline of the Incidence Rate Ratio for Major Bleeding as a Function of Continuous Duration of Concomitant Use of Selective Serotonin Reuptake Inhibitors and Oral Anticoagulants The shaded area indicates the upper and lower limits of the 95% CIs.

**Figure 2.**
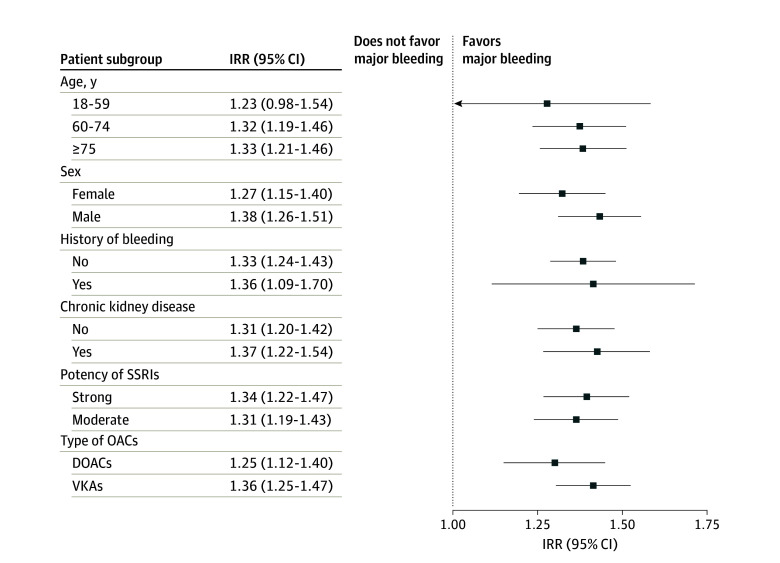
Results of Stratified Analyses for Major Bleeding Associated With Concomitant Use of Selective Serotonin Reuptake Inhibitors (SSRIs) and Oral Anticoagulants (OACs) Compared With OAC Use Alone DOAC indicates direct oral anticoagulant; IRR, incidence rate ratio; and VKA, vitamin K antagonist.

Concomitant use of SSRIs and OACs was associated with an increased risk of major bleeding compared with OAC use alone (IRR, 1.33; 95% CI, 1.24-1.42) (Table 2). The risk was the highest during the first 30 days of continuous use (IRR, 1.74; 95% CI, 1.37-2.22), and decreased thereafter (eTable 2 in Supplement 1). This trend was also observed when modeling the IRR flexibly as a spline of the duration of continuous use (Figure 1). The risk did not vary according to age, sex, history of major bleeding, chronic kidney disease (Figure 2; eTable 3 in Supplement 1), or potency of SSRIs (eTable 4 in Supplement 1). The risk of major bleeding was associated with concomitant use of SSRIs and DOACs compared with DOACs alone (IRR, 1.25; 95% CI, 1.12-1.40) and with concomitant use of SSRIs and VKAs compared with VKAs alone (IRR, 1.36; 95% CI, 1.25-1.47) (Table 3). With respect to types of major bleeding, the association was present for intracranial hemorrhage, gastrointestinal bleeding, and other major bleeding (Table 2). Last, concomitant use of SSRIs and OACs was also associated with the risk of any bleeding (IRR, 1.22; 95% CI, 1.16-1.28) compared with OAC use alone (eTable 5 in Supplement 1).

**Table 2.** Crude and Adjusted IRRs of Major Bleeding Associated With Concomitant Use of SSRIs and OACs, Overall and by Type of Bleeding^a^

Type of bleeding	No. (%) of participants		Crude IRR^c^	Adjusted IRR (95% CI)^d^
	Cases	Controls^b^		
Major bleeding	42 190	1 156 641		
OACs alone	31 417 (74.4)	881 988 (76.3)	1 [Reference]	1 [Reference]
SSRIs plus OACs	1127 (2.7)	21 708 (1.9)	1.43	1.33 (1.24-1.42)
Gastrointestinal bleeding	14 792	405 124		
OACs alone	10 690 (72.3)	310 256 (76.6)	1 [Reference]	1 [Reference]
SSRIs plus OACs	438 (3.0)	7768 (1.9)	1.60	1.38 (1.24-1.53)
Intracranial hemorrhage	5518	150 963		
OACs alone	3980 (72.1)	114 323 (75.7)	1 [Reference]	1 [Reference]
SSRIs plus OACs	170 (3.1)	3036 (2.0)	1.58	1.56 (1.32-1.85)
Other major bleeding	21 880	600 554		
OACs alone	16 747 (76.5)	457 409 (76.2)	1 [Reference]	1 [Reference]
SSRIs plus OACs	519 (2.4)	10 904 (1.8)	1.28	1.23 (1.12-1.36)

Abbreviations: IRR, incidence rate ratio; OACs, oral anticoagulants; SSRIs, selective serotonin reuptake inhibitors.

^a^ Use of SSRIs alone, non-SSRI antidepressants alone, multiple users, and nonusers were also included in the model for proper estimation of treatment effect.

^b^ Cases and controls were matched for age, sex, calendar year of cohort entry, and duration of follow-up.

^c^ IRR after matching of cases and controls.

^d^ Adjusted for all variables listed in Table 1.

**Table 3.** Crude and Adjusted IRRs of Major Bleeding Associated With Concomitant Use of SSRIs and OACs Compared With OAC Use Alone, According to Type of OAC

Type of OAC	No. (%) of participants		Crude IRR^b^	Adjusted IRR (95% CI)^c^
	Cases (n = 42 190)	Controls (n = 1 156 641)^a^		
DOACs^d^				
DOACs alone	9553 (22.6)	275 071 (23.8)	1 [Reference]	1 [Reference]
SSRIs plus DOACs	346 (0.8)	7235 (0.6)	1.35	1.25 (1.12-1.40)
VKAs^e^				
VKAs alone	21 864 (51.8)	606 917 (52.5)	1 [Reference]	1 [Reference]
SSRIs plus VKAs	781 (1.9)	14 473 (1.3)	1.47	1.36 (1.25-1.47)

Abbreviations: DOAC, direct oral anticoagulant; IRR, incidence rate ratio; OAC, oral anticoagulant; SSRI, selective serotonin reuptake inhibitor; VKAs, vitamin K antagonists.

^a^ Cases and controls were matched for age, sex, calendar year of cohort entry, and duration of follow-up.

^b^ IRR after matching of cases and controls.

^c^ Adjusted for all variables listed in Table 1.

^d^ Use of VKAs alone, SSRI plus VKAs, SSRIs alone, non-SSRI antidepressants alone, multiple users, and nonusers were also included in the model for proper estimation of treatment effect.

^e^ Use of DOACs alone, SSRI plus DOACs, SSRIs alone, non-SSRI antidepressants alone, multiple users, and nonusers were also included in the model for proper estimation of treatment effect.

In the assessment of interaction, a small superadditive interaction may have been present, although the estimate was not statistically significant (relative excess risk due to interaction [RERI], 0.10; 95% CI, −0.07 to 0.27) (eTable 6 in Supplement 1). Based on the estimated RERI, the interaction may be associated with approximately 5% of all major bleeding. In addition, there was limited evidence of a multiplicative interaction. Results from sensitivity analyses were consistent with those of the primary analysis (eTables 7-10 in Supplement 1). Finally, the association remained in the time-conditional propensity score–matched analysis, although slightly attenuated (adjusted hazard ratio, 1.23; 95% CI, 1.08-1.40) (eTable 11 in Supplement 1), and was consistent in the post hoc analysis (eTable 12 in Supplement 1).

## Discussion

In this population-based, nested case-control study, the concomitant use of SSRIs and OACs was associated with a 33% increased risk of major bleeding. The association was the strongest for the first few months of concomitant use. The overall risk remained consistent regardless of age, sex, potency of SSRIs, history of major bleeding, or chronic kidney disease as well as type of OAC. Concomitant use was individually associated with gastrointestinal bleeding, intracranial hemorrhage, and other major bleeding. Interaction between SSRIs and OACs, if any, was limited.

In light of these findings, the risk of major bleeding may be a pertinent safety consideration for patients using SSRIs and OACs concomitantly. This finding has been echoed in the summary of product characteristics for different OACs, which describes SSRIs as interacting drugs given that they independently increase the risk of bleeding. Although clinical guidelines for the management of major depressive disorder have acknowledged the risk of bleeding associated with SSRIs, the potential for interaction with OACs was either not discussed or based on very limited evidence.^49,50^ Likewise, guidelines from Canadian, US, and European cardiology associations for the management of AF suggest consideration of drug-drug interactions when prescribing OACs,^44,45,46^ with nonsteroidal anti-inflammatory drugs being the only class of drugs cited.^45^ Although the European Heart Rhythm Association lists SSRIs as drugs with pharmacodynamic interactions with DOACs, no evidence was cited.^51^

The risk of major bleeding associated with the concomitant use of SSRIs and OACs has been assessed in previous observational studies, although results were inconsistent.^7,17,18,19,20,22,23,24,52^ Limitations of previous studies included residual confounding,^19,20,22,24^ varying exposure definitions, limited statistical power,^20,22,23,24^ and the assessment of the concomitant use of SSRIs and OACs being a secondary objective.^7,18^ In line with our results, a systematic review and meta-analysis of 8 observational studies suggested an increased risk of major bleeding associated with the concomitant use of SSRIs and OACs (hazard ratio, 1.35; 95% CI, 1.14-1.58) compared with OAC use alone.^25^ However, several knowledge gaps were identified, including the risk of major bleeding in important patient subgroups. The present study confirmed that compared with OACs alone, concomitant use of SSRIs and OACs increased the risk of major bleeding among patients 60 years of age or older and among both sexes. One study previously assessed this association in these patient subgroups; however, statistical power was limited.^22^ Furthermore, we showed that the association remained similar irrespective of patients’ history of major bleeding or chronic kidney disease, both important factors in the HAS-BLED (Hypertension, Abnormal Renal/Liver Function, Stroke, Bleeding History or Predisposition, Labile International Normalized Ratio, Elderly [>65 Years], Drugs/Alcohol Concomitantly) score for major bleeding risk.^53^ Our findings also suggested that the concomitant use of SSRIs with both DOACs and VKAs was associated with an increased risk of major bleeding, with a possible lower risk with DOACs, although 95% CIs overlapped. A cohort study of patients from the ROCKET-AF (Rivaroxaban Once Daily Oral Direct Factor Xa Inhibition Compared With Vitamin K Antagonism for Prevention of Embolism and Stroke Trial in Atrial Fibrillation) trial suggested that the concomitant use of SSRIs and warfarin may increase the risk of major bleeding compared with rivaroxaban; however, the results were limited by high uncertainty and potential for selection bias and confounding.^20^ In another nested case-control study of nursing home residents, similar increases in risk were associated with the concomitant use of SSRIs and DOACs and of SSRIs and VKAs; however, statistical power was low.^23^

The increased risk of major bleeding with the concomitant use of SSRIs and OACs may occur through multiple mechanisms of action. During hemostasis, serotonin is released by platelets to enhance platelet activation and aggregation and prime them to interact with coagulation factors.^54^ Selective serotonin reuptake inhibitors block the serotonin reuptake transporter on platelet membranes and reduce serotonin content within platelets by up to 80% to 90%,^39,40^ decreasing the potency of hemostasis over time. In addition, some SSRIs, such as fluoxetine and fluvoxamine, inhibit the 1A2 and 2C9 isozymes of the hepatic cytochrome P450 enzyme, which play a key role in the metabolism of warfarin.^55^ Nonetheless, the interaction analysis suggests that the joint association of SSRIs and OACs is mainly owing to their individual risks of major bleeding; hence, any additional risk posed by pharmacokinetic interaction is likely minimal.

Although the increased risk of major bleeding does not suggest withholding treatment with either SSRIs or OACs, measures can be taken to mitigate this risk. Studies suggest that DOACs have lower potential for pharmacokinetic interactions with SSRIs than VKAs, and guidelines also recommend them over VKAs for the management of nonvalvular AF.^44,45,46,55,56^ Taken together with the findings in this study, DOACs may also be preferred for patients concomitantly using SSRIs. On the other hand, the risk of major bleeding was similar between SSRIs with more potent inhibition and SSRIs with less potent serotonin inhibition; thus; changing the SSRI may not be associated with bleeding risk. Finally, coprescription of proton pump inhibitors has also been suggested to prevent gastrointestinal bleeding.^51,57^ Overall, risk factors for bleeding should be monitored and managed to improve the safety of the concomitant use of SSRIs and OACs.^51^ Close monitoring is particularly essential within the first few months of concomitant use.

### Strengths and Limitations

This study has notable strengths. First, the selection of a large study population from routine care settings enhanced generalizability and provided sufficient statistical power to generate precise estimates in primary and secondary analyses. Second, selection bias was unlikely because we analyzed a well-defined cohort and used a nested case-control approach. Third, the assessment of additive and multiplicative interactions provided evidence suggesting that any biological interaction between use of SSRIs and OACs and the risk of major bleeding may only be marginally synergistic.^42^

This study also has some limitations. Residual confounding may affect the results given the observational nature of the study. The baseline risk for major bleeding may differ between patients concomitantly using SSRIs and those who were not. To mitigate potential bias, we adjusted for several potential confounders, including some lifestyle risk factors (such as BMI, smoking, and alcohol abuse). Furthermore, the results remained consistent in the time-conditional propensity score–matched analysis and in a post hoc analysis adjusted for additional comedications. Another consideration is that prescriptions recorded in the CPRD are those issued by general practitioners; hence, misclassification of exposure is possible if patients do not follow the treatment regimen. Prescriptions also do not include those issued by specialists, although AF as well as mild and moderate depression are managed mainly by general practitioners in the UK.^58,59^ To explore the potential for misclassification, we varied the exposure assessment window in sensitivity analyses, which produced results consistent with the main results. Finally, outcome misclassification through inaccurate recording of bleeding in the Hospital Episodes Statistics repository may occur. In addition, the physician’s judgment may be influenced by knowledge of patient treatment. To mitigate bias, we considered only primary diagnoses and did not include elective hospitalizations.

## Conclusions

In this large population-based, nested case-control study of patients with AF, the concomitant use of SSRIs and OACs was associated with an increased risk of major bleeding compared with OACs alone. To minimize the risk of bleeding, individual modifiable risk factors should be controlled, and patients should be closely monitored, particularly during the first few months of concomitant use.

## Notes

Supplement 1. **eMethods 1.** Interaction **eMethods 2.** Time-Conditional Propensity Score-Matched Analysis **eFigure.** Flowchart of Patient Selection in Study Cohort and Case-Control Selection **eTable 1.** *ICD-10* Codes Used to Define Major Bleeding **eTable 2.** Crude and Adjusted IRRs of Major Bleeding Associated With the Continuous Duration of Concomitant Use of SSRIs With OACs, Compared With OAC Use Alone **eTable 3.** Crude and Adjusted IRRs of Major Bleeding Associated With Concomitant Use of SSRIs With OACs, Stratified by Age, Sex, History of Bleeding, and History of Chronic Kidney Disease **eTable 4.** Crude and Adjusted IRRs of Major Bleeding Associated With Concomitant Use of Strong and Moderate SSRIs With OACs **eTable 5.** Crude and Adjusted IRRs of Any Bleeding Associated With Concomitant Use of SSRIs With OACs, Compared With OAC Use Alone **eTable 6.** Assessment of Additive and Multiplicative Interaction Between SSRIs and OACs, With Respect to Major Bleeding **eTable 7.** Crude and Adjusted IRRs of Major Bleeding Associated With Concomitant Use of SSRIs With OACs, Varying the Exposure Assessment Window **eTable 8.** Crude and Adjusted IRRs of Major Bleeding Associated With Concomitant Use of SSRIs With OACs, With Covariates Measured Prior to Cohort Entry **eTable 9.** Crude and Adjusted IRRs of Major Bleeding Associated With Concomitant Use of SSRIs With OACs, After Multiple Imputation of Missing BMI and Smoking Values **eTable 10.** Crude and Adjusted IRRs of Major Bleeding Associated With Concomitant Use of SSRIs With OACs, Compared With OAC Use Alone, by Type of OAC and Excluding Patients With Valvular AF **eTable 11.** Adjusted HRs of Major Bleeding Associated With Concomitant Use of SSRIs With OACs Compared With OAC Use Alone, in a Time-Conditional Propensity Score-Matched Analysis **eTable 12.** Crude and Adjusted IRRs of Major Bleeding Associated With Concomitant Use of SSRIs With OACs, With Adjustment for Additional Comedications Interacting With OACs
eReferences.

Supplement 2.
Data Sharing Statement

## References

[1] Brody DJ, Gu Q. Antidepressant Use Among Adults: United States, 2015-2018. National Center for Health Statistics; 2020.

[2] de Abajo FJ. Effects of selective serotonin reuptake inhibitors on platelet function: mechanisms, clinical outcomes and implications for use in elderly patients. Drugs Aging. 2011;28(5):345-367. doi:10.2165/11589340-000000000-00000 21542658

[3] Hillhouse TM, Porter JH. A brief history of the development of antidepressant drugs: from monoamines to glutamate. Exp Clin Psychopharmacol. 2015;23(1):1-21. doi:10.1037/a0038550 25643025 PMC4428540

[4] de Abajo FJ, Rodríguez LA, Montero D. Association between selective serotonin reuptake inhibitors and upper gastrointestinal bleeding: population based case-control study. BMJ. 1999;319(7217):1106-1109. doi:10.1136/bmj.319.7217.1106 10531103 PMC28262

[5] van Walraven C, Mamdani MM, Wells PS, Williams JI. Inhibition of serotonin reuptake by antidepressants and upper gastrointestinal bleeding in elderly patients: retrospective cohort study. BMJ. 2001;323(7314):655-658. doi:10.1136/bmj.323.7314.655 11566827 PMC55923

[6] Dalton SO, Johansen C, Mellemkjaer L, Nørgård B, Sørensen HT, Olsen JH. Use of selective serotonin reuptake inhibitors and risk of upper gastrointestinal tract bleeding: a population-based cohort study. Arch Intern Med. 2003;163(1):59-64. doi:10.1001/archinte.163.1.59 12523917

[7] Renoux C, Vahey S, Dell’Aniello S, Boivin JF. Association of selective serotonin reuptake inhibitors with the risk for spontaneous intracranial hemorrhage. JAMA Neurol. 2017;74(2):173-180. doi:10.1001/jamaneurol.2016.4529 27918771

[8] Movig KLL, Janssen MWHE, de Waal Malefijt J, Kabel PJ, Leufkens HGM, Egberts ACG. Relationship of serotonergic antidepressants and need for blood transfusion in orthopedic surgical patients. Arch Intern Med. 2003;163(19):2354-2358. doi:10.1001/archinte.163.19.2354 14581256

[9] Salkeld E, Ferris LE, Juurlink DN. The risk of postpartum hemorrhage with selective serotonin reuptake inhibitors and other antidepressants. J Clin Psychopharmacol. 2008;28(2):230-234. doi:10.1097/JCP.0b013e318166c52e 18344737

[10] Hackam DG, Mrkobrada M. Selective serotonin reuptake inhibitors and brain hemorrhage: a meta-analysis. Neurology. 2012;79(18):1862-1865. doi:10.1212/WNL.0b013e318271f848 23077009

[11] Laporte S, Chapelle C, Caillet P, . Bleeding risk under selective serotonin reuptake inhibitor (SSRI) antidepressants: a meta-analysis of observational studies. Pharmacol Res. 2017;118:19-32. doi:10.1016/j.phrs.2016.08.017 27521835

[12] Andrade C, Sandarsh S, Chethan KB, Nagesh KS. Serotonin reuptake inhibitor antidepressants and abnormal bleeding: a review for clinicians and a reconsideration of mechanisms. J Clin Psychiatry. 2010;71(12):1565-1575. doi:10.4088/JCP.09r05786blu 21190637

[13] Jiang HY, Chen HZ, Hu XJ, . Use of selective serotonin reuptake inhibitors and risk of upper gastrointestinal bleeding: a systematic review and meta-analysis. Clin Gastroenterol Hepatol. 2015;13(1):42-50.e3. doi:10.1016/j.cgh.2014.06.021 24993365

[14] Lindqvist PG, Nasiell J, Gustafsson LL, Nordstrom L. Selective serotonin reuptake inhibitor use during pregnancy increases the risk of postpartum hemorrhage and anemia: a hospital-based cohort study. J Thromb Haemost. 2014;12(12):1986-1992. doi:10.1111/jth.12757 25322909

[15] Douros A, Ades M, Renoux C. Risk of intracranial hemorrhage associated with the use of antidepressants inhibiting serotonin reuptake: a systematic review. CNS Drugs. 2018;32(4):321-334. doi:10.1007/s40263-018-0507-7 29536379

[16] Baumgartner C, Fan D, Fang MC, . Anxiety, depression, and adverse clinical outcomes in patients with atrial fibrillation starting warfarin: Cardiovascular Research Network WAVE Study. J Am Heart Assoc. 2018;7(8):e007814. doi:10.1161/JAHA.117.007814 29656278 PMC6015441

[17] Kurdyak PA, Juurlink DN, Kopp A, Herrmann N, Mamdani MM. Antidepressants, warfarin, and the risk of hemorrhage. J Clin Psychopharmacol. 2005;25(6):561-564. doi:10.1097/01.jcp.0000186869.67418.bc 16282838

[18] Komen JJ, Hjemdahl P, Mantel-Teeuwisse AK, Klungel OH, Wettermark B, Forslund T. Concomitant anticoagulant and antidepressant therapy in atrial fibrillation patients and risk of stroke and bleeding. Clin Pharmacol Ther. 2020;107(1):287-294. doi:10.1002/cpt.1603 31506933

[19] Schelleman H, Brensinger CM, Bilker WB, Hennessy S. Antidepressant-warfarin interaction and associated gastrointestinal bleeding risk in a case-control study. PLoS One. 2011;6(6):e21447. doi:10.1371/journal.pone.0021447 21731754 PMC3123326

[20] Quinn GR, Hellkamp AS, Hankey GJ, . Selective serotonin reuptake inhibitors and bleeding risk in anticoagulated patients with atrial fibrillation: an analysis from the ROCKET AF trial. J Am Heart Assoc. 2018;7(15):e008755. doi:10.1161/JAHA.118.008755 30371223 PMC6201450

[21] Cochran KA, Cavallari LH, Shapiro NL, Bishop JR. Bleeding incidence with concomitant use of antidepressants and warfarin. Ther Drug Monit. 2011;33(4):433-438. doi:10.1097/FTD.0b013e318224996e 21743381 PMC3212440

[22] Lee MT, Park KY, Kim MS, You SH, Kang YJ, Jung SY. Concomitant use of NSAIDs or SSRIs with NOACs requires monitoring for bleeding. Yonsei Med J. 2020;61(9):741-749. doi:10.3349/ymj.2020.61.9.741 32882758 PMC7471076

[23] Jobski K, Hoffmann F, Herget-Rosenthal S, Dörks M. Drug interactions with oral anticoagulants in German nursing home residents: comparison between vitamin K antagonists and non–vitamin K antagonist oral anticoagulants based on two nested case-control studies. Clin Res Cardiol. 2020;109(4):465-475. doi:10.1007/s00392-019-01526-7 31286199

[24] Zhang Y, Souverein PC, Gardarsdottir H, van den Ham HA, Maitland-van der Zee AH, de Boer A. Risk of major bleeding among users of direct oral anticoagulants combined with interacting drugs: a population-based nested case-control study. Br J Clin Pharmacol. 2020;86(6):1150-1164. doi:10.1111/bcp.14227 32022295 PMC7256117

[25] Rahman AA, He N, Rej S, Platt RW, Renoux C. Concomitant use of selective serotonin reuptake inhibitors and oral anticoagulants and risk of major bleeding: a systematic review and meta-analysis. Thromb Haemost. 2023;123(1):54-63. doi:10.1055/a-1932-8976 36037829

[26] Herrett E, Gallagher AM, Bhaskaran K, . Data resource profile: Clinical Practice Research Datalink (CPRD). Int J Epidemiol. 2015;44(3):827-836. doi:10.1093/ije/dyv098 26050254 PMC4521131

[27] Wolf A, Dedman D, Campbell J, . Data resource profile: Clinical Practice Research Datalink (CPRD) Aurum. Int J Epidemiol. 2019;48(6):1740-1740g. doi:10.1093/ije/dyz034 30859197 PMC6929522

[28] Herbert A, Wijlaars L, Zylbersztejn A, Cromwell D, Hardelid P. Data resource profile: Hospital Episode Statistics Admitted Patient Care (HES APC). Int J Epidemiol. 2017;46(4):1093-1093i. doi:10.1093/ije/dyx015 28338941 PMC5837677

[29] Office for National Statistics. What we do. Accessed April 15, 2023. https://www.ons.gov.uk/aboutus/whatwedo

[30] von Elm E, Altman DG, Egger M, Pocock SJ, Gøtzsche PC, Vandenbroucke JP; STROBE Initiative. The Strengthening the Reporting of Observational Studies in Epidemiology (STROBE) statement: guidelines for reporting observational studies. Epidemiology. 2007;18(6):800-804. doi:10.1097/EDE.0b013e3181577654 18049194

[31] Essebag V, Genest J Jr, Suissa S, Pilote L. The nested case-control study in cardiology. Am Heart J. 2003;146(4):581-590. doi:10.1016/S0002-8703(03)00512-X 14564310

[32] Crooks CJ, Card TR, West J. Defining upper gastrointestinal bleeding from linked primary and secondary care data and the effect on occurrence and 28 day mortality. BMC Health Serv Res. 2012;12(392):392. doi:10.1186/1472-6963-12-392 23148590 PMC3531298

[33] Burns EM, Rigby E, Mamidanna R, . Systematic review of discharge coding accuracy. J Public Health (Oxf). 2012;34(1):138-148. doi:10.1093/pubmed/fdr054 21795302 PMC3285117

[34] Shehab N, Ziemba R, Campbell KN, . Assessment of *ICD-10-CM* code assignment validity for case finding of outpatient anticoagulant-related bleeding among Medicare beneficiaries. Pharmacoepidemiol Drug Saf. 2019;28(7):951-964. doi:10.1002/pds.4783 31144403 PMC13086182

[35] Schneeweiss S, Suissa S. Advanced approaches to controlling confounding in pharmacoepidemiologic studies. In: Strom BL, Kimmel SE, Hennessy S, eds. Pharmacoepidemiology. 6th ed. John Wiley & Sons, Ltd; 2020.

[36] Essebag V, Platt RW, Abrahamowicz M, Pilote L. Comparison of nested case-control and survival analysis methodologies for analysis of time-dependent exposure. BMC Med Res Methodol. 2005;5(1):5. doi:10.1186/1471-2288-5-5 15670334 PMC548149

[37] Mahadevan P, Harley M, Fordyce S, . Completeness and representativeness of small area socioeconomic data linked with the UK Clinical Practice Research Datalink (CPRD). J Epidemiol Community Health. 2022;76(10):880-886. doi:10.1136/jech-2022-219200 35902219 PMC9484378

[38] Tatsumi M, Groshan K, Blakely RD, Richelson E. Pharmacological profile of antidepressants and related compounds at human monoamine transporters. Eur J Pharmacol. 1997;340(2-3):249-258. doi:10.1016/S0014-2999(97)01393-9 9537821

[39] Hergovich N, Aigner M, Eichler HG, Entlicher J, Drucker C, Jilma B. Paroxetine decreases platelet serotonin storage and platelet function in human beings. Clin Pharmacol Ther. 2000;68(4):435-442. doi:10.1067/mcp.2000.110456 11061584

[40] Javors MA, Houston JP, Tekell JL, Brannan SK, Frazer A. Reduction of platelet serotonin content in depressed patients treated with either paroxetine or desipramine. Int J Neuropsychopharmacol. 2000;3(3):229-235. doi:10.1017/S146114570000198X 11343600

[41] VanderWeele TJ, Knol MJ. A tutorial on interaction. Epidemiol Methods. 2014;3(1):33-72. doi:10.1515/em-2013-0005

[42] Rothman KJ. Epidemiology: An Introduction. 2nd ed. Oxford University Press, Inc; 2012.

[43] Raghunathan TE, Lepkowski JM, Van Hoewyk J, Solenberger P. A multivariate technique for multiply imputing missing values using a sequence of regression models. *Surv Methodol*. 2001;27(1):85-95.

[44] Hindricks G, Potpara T, Dagres N, ; ESC Scientific Document Group. 2020 ESC guidelines for the diagnosis and management of atrial fibrillation developed in collaboration with the European Association for Cardio-Thoracic Surgery (EACTS): the Task Force for the Diagnosis and Management of Atrial Fibrillation of the European Society of Cardiology (ESC) developed with the special contribution of the European Heart Rhythm Association (EHRA) of the ESC. Eur Heart J. 2021;42(5):373-498. doi:10.1093/eurheartj/ehaa612 32860505

[45] January CT, Wann LS, Calkins H, . 2019 AHA/ACC/HRS focused update of the 2014 AHA/ACC/HRS Guideline for the Management of Patients With Atrial Fibrillation: a report of the American College of Cardiology/American Heart Association Task Force on Clinical Practice Guidelines and the Heart Rhythm Society in Collaboration With the Society of Thoracic Surgeons. Circulation. 2019;140(2):e125-e151. doi:10.1161/CIR.0000000000000665 30686041

[46] Andrade JG, Aguilar M, Atzema C, ; Members of the Secondary Panel. The 2020 Canadian Cardiovascular Society/Canadian Heart Rhythm Society comprehensive guidelines for the management of atrial fibrillation. Can J Cardiol. 2020;36(12):1847-1948. doi:10.1016/j.cjca.2020.09.001 33191198

[47] Suissa S, Moodie EE, Dell’Aniello S. Prevalent new-user cohort designs for comparative drug effect studies by time-conditional propensity scores. Pharmacoepidemiol Drug Saf. 2017;26(4):459-468. doi:10.1002/pds.4107 27610604

[48] Suissa S, Dell’Aniello S, Renoux C. The prevalent new-user design for studies with no active comparator: the example of statins and cancer. Epidemiology. 2023;34(5):681-689. doi:10.1097/EDE.0000000000001628 37195285

[49] Kennedy SH, Lam RW, McIntyre RS, ; CANMAT Depression Work Group. Canadian Network for Mood and Anxiety Treatments (CANMAT) 2016 clinical guidelines for the management of adults with major depressive disorder, section 3: pharmacological treatments. Can J Psychiatry. 2016;61(9):540-560. doi:10.1177/0706743716659417 27486148 PMC4994790

[50] Gelenberg AJ, Freeman MP, Markowitz JC, . Practice guideline for the treatment of patients with major depressive disorder. American Psychiatric Association. 2010. Accessed April 30, 2023. https://psychiatryonline.org/pb/assets/raw/sitewide/practice_guidelines/guidelines/mdd.pdf

[51] Steffel J, Collins R, Antz M, ; External reviewers. 2021 European Heart Rhythm Association practical guide on the use of non–vitamin K antagonist oral anticoagulants in patients with atrial fibrillation. Europace. 2021;23(10):1612-1676. doi:10.1093/europace/euab065 33895845 PMC11636576

[52] Schalekamp T, Klungel OH, Souverein PC, de Boer A. Increased bleeding risk with concurrent use of selective serotonin reuptake inhibitors and coumarins. Arch Intern Med. 2008;168(2):180-185. doi:10.1001/archinternmed.2007.32 18227365

[53] Pisters R, Lane DA, Nieuwlaat R, de Vos CB, Crijns HJ, Lip GY. A novel user-friendly score (HAS-BLED) to assess 1-year risk of major bleeding in patients with atrial fibrillation: the Euro Heart Survey. Chest. 2010;138(5):1093-1100. doi:10.1378/chest.10-0134 20299623

[54] Halperin D, Reber G. Influence of antidepressants on hemostasis. Dialogues Clin Neurosci. 2007;9(1):47-59. doi:10.31887/DCNS.2007.9.1/dhalperin 17506225 PMC3181838

[55] Sansone RA, Sansone LA. Warfarin and antidepressants: happiness without hemorrhaging. Psychiatry (Edgmont). 2009;6(7):24-29. 19724766 PMC2728939

[56] Galgani A, Palleria C, Iannone LF, . Pharmacokinetic interactions of clinical interest between direct oral anticoagulants and antiepileptic drugs. Front Neurol. 2018;9:1067. doi:10.3389/fneur.2018.01067 30581412 PMC6292857

[57] Ray WA, Chung CP, Murray KT, . Association of oral anticoagulants and proton pump inhibitor cotherapy with hospitalization for upper gastrointestinal tract bleeding. JAMA. 2018;320(21):2221-2230. doi:10.1001/jama.2018.17242 30512099 PMC6404233

[58] National Institute for Health and Care Excellence. Depression in adults: treatment and management. 2022. Accessed April 30, 2023. https://www.nice.org.uk/guidance/ng222 35977056

[59] Adderley NJ, Ryan R, Nirantharakumar K, Marshall T. Prevalence and treatment of atrial fibrillation in UK general practice from 2000 to 2016. Heart. 2019;105(1):27-33. doi:10.1136/heartjnl-2018-312977 29991504

