# Supplementary material for: Concomitant Use of Selective Serotonin Reuptake Inhibitors With Oral Anticoagulants and Risk of Major Bleeding
Source: JAMA Netw Open. 2024 Mar 22;7(3):e243208. doi: 10.1001/jamanetworkopen.2024.3208 (PMC10960200; doi:10.1001/jamanetworkopen.2024.3208)
Supplement: Supplement 1. — eMethods 1. Interaction eMethods 2. Time-Conditional Propensity Score-Matched Analysis eFigure. Flowchart of Patient Selection in Study Cohort and Case-Control Selection eTable 1. ICD-10 Codes Used to Define Major Bleeding eTable 2. Crude and Adjusted IRRs of Major Bleeding Associated With the Continuous Duration of Concomitant Use of SSRIs With OACs, Compared With OAC Use Alone eTable 3. Crude and Adjusted IRRs of Major Bleeding Associated With Concomitant Use of SSRIs With OACs, Stratified by Age, Sex, History of Bleeding, and History of Chronic Kidney Disease eTable 4. Crude and Adjusted IRRs of Major Bleeding Associated With Concomitant Use of Strong and Moderate SSRIs With OACs eTable 5. Crude and Adjusted IRRs of Any Bleeding Associated With Concomitant Use of SSRIs With OACs, Compared With OAC Use Alone eTable 6. Assessment of Additive and Multiplicative Interaction Between SSRIs and OACs, With Respect to Major Bleeding eTable 7. Crude and Adjusted IRRs of Major Bleeding Associated With Concomitant Use of SSRIs With OACs, Varying the Exposure Assessment Window eTable 8. Crude and Adjusted IRRs of Major Bleeding Associated With Concomitant Use of SSRIs With OACs, With Covariates Measured Prior to Cohort Entry eTable 9. Crude and Adjusted IRRs of Major Bleeding Associated With Concomitant Use of SSRIs With OACs, After Multiple Imputation of Missing BMI and Smoking Values eTable 10. Crude and Adjusted IRRs of Major Bleeding Associated With Concomitant Use of SSRIs With OACs, Compared With OAC Use Alone, by Type of OAC and Excluding Patients With Valvular AF eTable 11. Adjusted HRs of Major Bleeding Associated With Concomitant Use of SSRIs With OACs Compared With OAC Use Alone, in a Time-Conditional Propensity Score-Matched Analysis eTable 12. Crude and Adjusted IRRs of Major Bleeding Associated With Concomitant Use of SSRIs With OACs, With Adjustment for Additional Comedications Interacting With OACs eReferences. [file jamanetwopen-e243208-s001.pdf]

Supplementary Online Content

Rahman AA, Platt RW, Beradid S, Boivin JF, Rej S, Renoux C. Concomitant use of selective serotonin reuptake inhibitors with oral anticoagulants and risk of major bleeding. *JAMA Netw Open.* 2024;7(3):e243208.
doi:10.1001/jamanetworkopen.2024.3208

eMethods 1. Interaction
eMethods 2. Time-Conditional Propensity Score-Matched Analysis
eFigure. Flowchart of Patient Selection in Study Cohort and Case-Control Selection
eTable 1. *ICD-10* Codes Used to Define Major Bleeding
eTable 2. Crude and Adjusted IRRs of Major Bleeding Associated With the Continuous Duration of Concomitant Use of SSRIs With OACs, Compared With OAC Use Alone
eTable 3. Crude and Adjusted IRRs of Major Bleeding Associated With Concomitant Use of SSRIs With OACs, Stratified by Age, Sex, History of Bleeding, and History of Chronic Kidney Disease
eTable 4. Crude and Adjusted IRRs of Major Bleeding Associated With Concomitant Use of Strong and Moderate SSRIs With OACs
eTable 5. Crude and Adjusted IRRs of Any Bleeding Associated With Concomitant Use of SSRIs With OACs, Compared With OAC Use Alone
eTable 6. Assessment of Additive and Multiplicative Interaction Between SSRIs and OACs, With Respect to Major Bleeding
eTable 7. Crude and Adjusted IRRs of Major Bleeding Associated With Concomitant Use of SSRIs With OACs, Varying the Exposure Assessment Window
eTable 8. Crude and Adjusted IRRs of Major Bleeding Associated With Concomitant Use of SSRIs With OACs, With Covariates Measured Prior to Cohort Entry
eTable 9. Crude and Adjusted IRRs of Major Bleeding Associated With Concomitant Use of SSRIs With OACs, After Multiple Imputation of Missing BMI and Smoking Values
eTable 10. Crude and Adjusted IRRs of Major Bleeding Associated With Concomitant Use of SSRIs With OACs, Compared With OAC Use Alone, by Type of OAC and Excluding Patients With Valvular AF
eTable 11. Adjusted HRs of Major Bleeding Associated With Concomitant Use of SSRIs With OACs Compared With OAC Use Alone, in a Time-Conditional Propensity Score-Matched Analysis
eTable 12. Crude and Adjusted IRRs of Major Bleeding Associated With Concomitant Use of SSRIs With OACs, With Adjustment for Additional Comedications Interacting With OACs
eReferences.

This supplementary material has been provided by the authors to give readers additional information about their work.

eMethods 1. Interaction

We assessed whether an interaction was present between SSRIs and OACs with respect to the risk of major bleeding on both the additive and multiplicative scales, in other words, whether the joint effect of the two exposures departed from the sum or product of their individual effects on the risk of bleeding. Though, additive interaction has been described as most indicative of biological or mechanistic interaction [1, 2]. Patients were classified into one of six mutually exclusive exposure categories: concomitant use of SSRIs and OACs, SSRI use alone, OAC use alone, other use, past use, and non-use. We defined users of SSRIs and/or OACs as patients whose last prescription covered or ended 30 days before the index date. Past users included patients whose last prescription for a medication of interest ended within 31 days to 365 days (one year) of the index date. Non-users did not have any prescriptions for study medications of interest in the year before the index date. With non-use as a reference category, we estimated RRs for major bleeding associated with concomitant use of SSRIs and OACs, SSRI use alone, and OAC use alone. To assess additive interaction, we determined the relative excess risk due to interaction (RERI) [1, 2]. The RERI can be calculated using the IRRs associated with concomitant use of SSRIs and OACs, OACs alone, and SSRIs alone, compared with non-use, as $IRR_{11} – IRR_{10} – IRR_{01} + 1$ [1, 2]. An additive interaction is present if the RERI does not equal zero [1, 2]. We also assessed the attributable proportion of disease as $RERI / IRR_{11}$, which expresses the proportion of disease due to the additive interaction [1, 2]. We also determined the multiplicative interaction IRR, computed as $IRR_{11} / (IRR_{01} \times IRR_{10})$. Multiplicative interaction is present if the value of the IRR did not equal one.

© 2024 Rahman AA et al. *JAMA Network Open.*

eMethods 2. Time-Conditional Propensity Score-Matched Analysis

We conducted a supplementary time-conditional propensity score (TCPS)-matched analysis to further explore the potential for residual confounding [3, 4]. Briefly, among the base cohort of patients with incident AF initiating OACs, we identified each patient initiating SSRIs to generate time-based exposure sets that included all potential comparators using OACs alone up to that point in time with the same age (± 1 year), sex, and calendar date of OAC initiation (± 1 year). Next, starting chronologically, each concomitant user was matched 1:1 without replacement to a patient using OACs alone with the closest TCPS in the corresponding exposure set, after trimming within each set to meet the positivity assumption (avoiding extrapolation of data with no comparable treated or untreated patients). The TCPS included the same time-varying patient characteristics used in the primary analysis, measured on or before study cohort entry. For each of the matched pairs, study cohort entry was defined as the date of SSRI initiation for concomitant users and the date that resulted in the same time since OAC initiation in those using OAC alone. Patients were followed up until hospitalization for major bleeding, discontinuation of SSRIs for concomitant users, initiation of SSRIs for those using OACs alone, discontinuation of OACs, death from any cause, end of registration with the practice, or end of the study period, whichever occurred first. We used a Cox proportional hazards model with robust standard errors to estimate the hazard ratio (HR) and 95% CI of major bleeding associated with concomitant use of SSRIs and OACs, compared with OAC use alone.

© 2024 Rahman AA et al. *JAMA Network Open.*

eFigure. Flowchart of Patient Selection in Study Cohort and Case-Control Selection

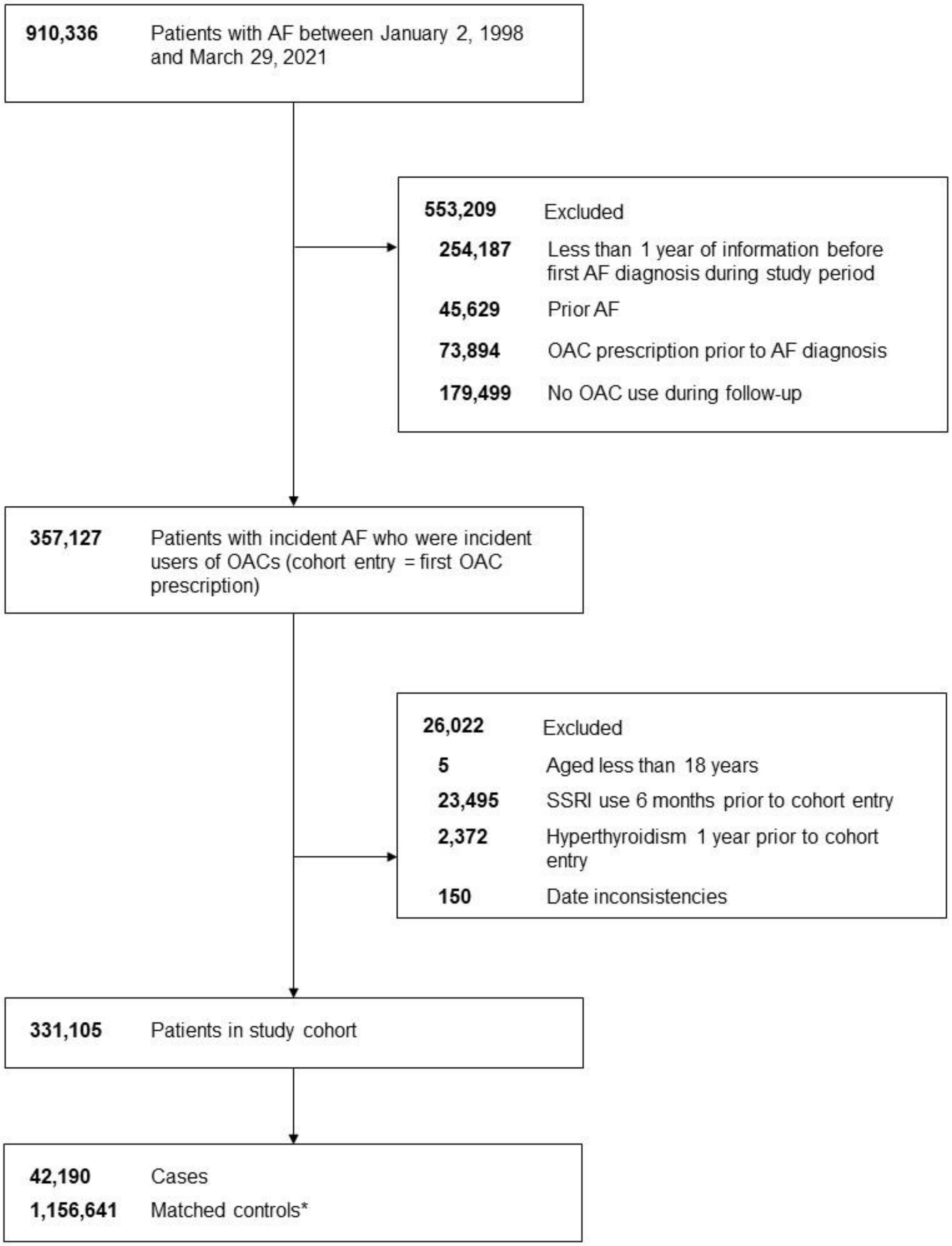

*Cases were defined as patients with a first hospitalization with a primary diagnosis of or death with bleeding as the primary cause. For each case, we randomly selected up to 30 controls among the cohort members from the risk-sets defined by the case, matched on age, sex, calendar date of cohort entry (± 6 months), and duration of follow-up.

© 2024 Rahman AA et al. *JAMA Network Open.*

eTable 1. *ICD-10* Codes Used to Define Major Bleeding

**ICD-10**	**Label**
**Gastrointestinal bleeding**	
I85.0	Oesphageal varices with bleeding
I98.3	Oesphageal varices with bleeding in diseases classified elsewhere (overlapping codes)
K22.11	Ulcer of esophagus with bleeding
K22.6	Gastro-oesophageal laceration-hemorrhage syndrome/Mallory-Weiss syndrome
K22.8	Other specified disease of esophagus, hemorrhage of esophagus NOS
K25.0, K25.2, K25.4, K25.6	Gastric ulcer, acute or chronic, with hemorrhage and/or perforation
K26.0, K26.2, K26.4, K26.6	Duodenal ulcer, acute or chronic, with hemorrhage and/or perforation
K27.0, K27.2, K27.4, K27.6	Peptic ulcer (site unspecified), acute or chronic, with hemorrhage and/or perforation
K28.0, K28.2, K28.4, K28.6	Gastrojejunal ulcer (site unspecified), acute or chronic, with hemorrhage and/or perforation
K29.0	Acute hemorrhagic gastritis
K29.21	Alcoholic gastritis (with hemorrhage)
K29.61	Other gastritis with bleeding
K29.71	Gastritis (unspecified with bleeding)
K29.81	Duodenitis (with hemorrhage)
K29.91	Gastroduodenitis (unspecified with bleeding)
K31.81	Angiodysplasia of stomach and duodenum with bleeding
K31.82	Dieulafoy lesion (hemorrhagic) of stomach and duodenum
K55.21	Angiodysplasia of colon with bleeding
K57.11	Diverticulosis of small intestine without perforation or abscess with bleeding
K57.13	Diverticulitis of small intestine without perforation or abscess with bleeding
K57.31	Diverticulosis of large intestine without perforation or abscess with bleeding
K57.33	Diverticulitis of large intestine without perforation or abscess with bleeding
K57.53	Diverticulitis of both small and large intestine without perforation or abscess with bleeding
K57.93	Diverticulitis of intestine, part unspecified, without perforation or abscess with bleeding
K62.5	Hemorrhage of anus and rectum
K63.81	Dieulafoy lesion (hemorrhagic) of colon
K66.1	Hemoperitoneum
K92.0	Hematemesis
K92.1	Melena
K92.2	GI hemorrhage, unspecified
**Intracranial hemorrhage**	

© 2024 Rahman AA et al. *JAMA Network Open.*

I60.x	Subarachnoid haemorrhage
I61.x	Intracerebral haemorrhage
I62.x	Other nontraumatic intracranial haemorrhage
**Other major bleeding**	
D68.3	Hemorrhagic disorder due to circulating anticoagulants
D69.9	Unspecified hemorrhagic conditions
H11.3	Conjunctival hemorrhage
H21.0	Hyphema
H31.3	Choroidal hemorrhage and rupture
H35.6	Retinal hemorrhage
H43.1	Vitreous hemorrhage
H45.0	Vitreous hemorrhage in diseases classified elsewhere
H92.2	Otorrhagia
I23.0	Hemopericardium as current complication following acute myocardial infarction
I31.2	Hemopericardium NEC
J94.2	Haemothorax
M25.0	Hemarthrosis
N02.x	Recurrent and persistent hematuria
N42.1	Other disorders of the prostate: Congestion and hemorrhage of prostate
N83.6	Hematosalpinx
N83.7	Hematoma of broad ligament
N85.7	Hematometra
N89.7	Hematocolpos
N92.0, N92.1, N92.2, N92.4	Excessive, frequent and irregular menstruation (with regular cycle, irregular cycle, puberty, premenopausal period)
N93.8, N93.9	Other specified and unspecified abnormal uterine and vaginal bleeding
N95.0	Postmenopausal bleeding
R04.x	Hemorrhage from respiratory passages (epistaxis, hemorrhage from throat, hemoptysis, from other sites in passages, unspecified from respiratory passages)
R31.x	Hematuria (gross, benign, other, unspecified)
R58	Hemorrhage, not elsewhere classified

© 2024 Rahman AA et al. *JAMA Network Open.*

eTable 2. Crude and Adjusted IRRs of Major Bleeding Associated With the Continuous Duration of Concomitant Use of SSRIs With OACs, Compared With OAC Use Alone.[a]

	No. (%) of Participants			
	**Cases (n=15,157)**	**Controls[b] (n=395,330)**	**Crude RR[c]**	**Adjusted IRR[d] (95% CI)**
OACs alone	14,813 (97.7)	389,435 (98.5)	1 [Reference]	1 [Reference]
SSRIs + OACs	344 (2.3)	5,895 (1.5)	1.42	1.29 (1.15-1.45)
≤ 30 days	74 (0.5)	1,006 (0.3)	1.89	1.74 (1.37-2.22)
31-180 days	131 (0.9)	2,346 (0.6)	1.44	1.31 (1.10-1.58)
> 180 days	139 (0.9)	2,543 (0.6)	1.22	1.11 (0.93-1.33)

CI = confidence interval, IRR = incidence rate ratio, OAC = oral anticoagulant, SSRI = selective serotonin reuptake inhibitor

a. Patients censored upon discontinuation of OACs.

b. Cases and controls were matched for age, sex, calendar year of cohort entry, and duration of follow-up.

c. IRR after matching of cases and controls.

d. Adjusted for all variables listed in Table 1.

© 2024 Rahman AA et al. *JAMA Network Open.*

eTable 3. Crude and Adjusted IRRs of Major Bleeding Associated With Concomitant Use of SSRIs With OACs, Stratified by Age, Sex, History of Bleeding, and History of Chronic Kidney Disease.[a]

	No. (%) of Participants			
	**Cases**	**Controls[b]**	**Crude IRR[c]**	**Adjusted IRR[d] (95% CI)**
**Sex**				
**Female**	**16,979**	**459,262**		
OACs alone	12,428 (73.2)	344,594 (75.0)	1 [Reference]	1 [Reference]
SSRIs + OACs	547 (3.2)	11,031 (2.4)	1.35	1.27 (1.15-1.39)
**Male**	**25,211**	**697,379**		
OACs alone	18,989 (75.3)	537,394 (77.1)	1 [Reference]	1 [Reference]
SSRIs + OACs	580 (2.3)	10,677 (1.5)	1.50	1.38 (1.26-1.51)
**Age**				
**18-59 years**	**2,952**	**64,921**		
OACs alone	1,982 (67.1)	42,164 (65.0)	1 [Reference]	1 [Reference]
SSRIs + OACs	105 (3.6)	1,372 (2.1)	1.47	1.23 (0.98-1.54)
**60-74 years**	**16,973**	**473,366**		
OACs alone	12,716 (74.9)	360,167 (76.1)	1 [Reference]	1 [Reference]
SSRIs + OACs	480 (2.8)	9,052 (1.9)	1.49	1.32 (1.19-1.46)
**≥ 75 years**	**22,265**	**618,354**		
OACs alone	16,719 (75.1)	479,657 (77.6)	1 [Reference]	1 [Reference]
SSRIs + OACs	542 (2.4)	11,284 (1.8)	1.37	1.33 (1.21-1.46)
**History of bleeding**				
**No**	**36,152**	**898,241**		
OACs alone	26,874 (74.3)	680,815 (75.8)	1 [Reference]	1 [Reference]
SSRIs + OACs	974 (2.7)	16,954 (1.9)	1.44	1.33 (1.24-1.43)
**Yes**	**6,038**	**18,347**		
OACs alone	4,543 (75.2)	14,318 (78.0)	1 [Reference]	1 [Reference]
SSRIs + OACs	153 (2.5)	350 (1.9)	1.36	1.36 (1.09-1.70)
**History of chronic kidney disease**				
**No**	**29,564**	**617,705**		
OACs alone	22,184 (75.0)	472,133 (76.4)	1 [Reference]	1 [Reference]
SSRIs + OACs	732 (2.5)	10,240 (1.7)	1.43	1.31 (1.20-1.42)
**Yes**	**12,626**	**119,261**		
OACs alone	9,233 (73.1)	89,185 (74.8)	1 [Reference]	1 [Reference]
SSRIs + OACs	395 (3.1)	2,846 (2.4)	1.45	1.37 (1.22-1.54)

CI = confidence interval, IRR = incidence rate ratio, OAC = oral anticoagulant, SSRI = selective serotonin reuptake inhibitor
a. Use of SSRIs alone, non-SSRI antidepressants alone, multiple users, and non-users were also included in the model for proper estimation of treatment effect.
b. Cases and controls were matched for age, sex, calendar year of cohort entry, and duration of follow-up.
c. IRR after matching of cases and controls.
d. Adjusted for all variables listed in Table 1.

eTable 4. Crude and Adjusted IRRs of Major Bleeding Associated With Concomitant Use of Strong and Moderate SSRIs With OACs.[a]

	No. (%) of Participants			
	**Cases (n=42,190)**	**Controls[b] (n=1,156,641)**	**Crude IRR[c]**	**Adjusted IRR[d] (95% CI)**
OACs alone	31,417 (74.4)	881,988 (76.3)	1 [Reference]	1 [Reference]
Strong SSRIs[e] + OACs	541 (1.3)	10,273 (0.9)	1.46	1.34 (1.22-1.47)
Moderate SSRIs[f] + OACs	586 (1.4)	11,435 (1.0)	1.41	1.31 (1.19-1.43)

CI = confidence interval, IRR = incidence rate ratio, OAC = oral anticoagulant, SSRI = selective serotonin reuptake inhibitor
a. Use of SSRIs alone, non-SSRI antidepressants alone, multiple users, and non-users were also included in the model for proper estimation of treatment effect.
b. Cases and controls were matched for age, sex, calendar year of cohort entry, and duration of follow-up.
c. IRR after matching of cases and controls.
d. Adjusted for all variables listed in Table 1.
e. Includes fluoxetine, paroxetine, and sertraline.
f. Includes citalopram, escitalopram, and fluvoxamine.

© 2024 Rahman AA et al. *JAMA Network Open.*

eTable 5. Crude and Adjusted IRRs of Any Bleeding Associated With Concomitant Use of SSRIs With OACs, Compared With OAC Use Alone.[a]

	No. (%) of Participants			
	Cases (n=76,271)	Controls[b] (n=2,082,657)	Crude IRR[c]	Adjusted IRR[d] (95% CI)
OACs alone	59,252 (77.7)	1,615,860 (77.6)	1 [Reference]	1 [Reference]
SSRIs + OACs	1,852 (2.4)	36,261 (1.7)	1.34	1.22 (1.16-1.28)

CI = confidence interval, IRR = incidence rate ratio, OAC = oral anticoagulant, SSRI = selective serotonin reuptake inhibitor
a. Use of SSRIs alone, non-SSRI antidepressants alone, multiple users, and non-users were also included in the model for proper estimation of treatment effect.
b. Cases and controls were matched for age, sex, calendar year of cohort entry, and duration of follow-up.
c. IRR after matching of cases and controls.
d. Adjusted for all variables listed in Table 1.

© 2024 Rahman AA et al. *JAMA Network Open.*

eTable 6. Assessment of Additive and Multiplicative Interaction Between SSRIs and OACs, With Respect to Major Bleeding.[a]

	No. (%) of Participants			
	Cases (n=60,128)	Controls[b] (n=1,696,204)	Crude IRR[c]	Adjusted IRR[d] (95% CI)
No use	18,198 (30.3)	637,286 (37.6)	1 [Reference]	1 [Reference]
OACs alone	30,818 (51.3)	809,755 (47.7)	1.45	1.49 (1.46-1.53)
SSRIs alone	1,080 (1.8)	24,672 (1.5)	1.52	1.40 (1.31-1.50)
SSRIs + OACs	1,286 (2.1)	23,527 (1.4)	2.07	1.99 (1.86-2.12)
**RERI (95% CI)**				0.10 (-0.07-0.27)
**Attributable proportion of disease (%)**				5.0
**Multiplicative interaction OR (95% CI)**				0.95 (0.73-1.17)

CI = confidence interval, IRR = incidence rate ratio, OAC = oral anticoagulant, RERI = relative excess risk due to interaction, SSRI = selective serotonin reuptake inhibitor

a. Use of non-SSRI antidepressants alone and concomitantly with OACs and/or SSRIs as well as past use of any study medications were included in the model for proper estimation of treatment effect.

b. Cases and controls were matched for age, sex, calendar year of cohort entry, and duration of follow-up.

c. IRR after matching of cases and controls.

d. Adjusted for all variables listed in Table 1.

© 2024 Rahman AA et al. *JAMA Network Open.*

eTable 7. Crude and Adjusted IRRs of Major Bleeding Associated With Concomitant Use of SSRIs With OACs, Varying the Exposure Assessment Window.[a]

	No. (%) of Participants			
	Cases (n=42,190)	Controls[b] (n=1,156,641)	Crude IRR[c]	Adjusted IRR[d] (95% CI)
**Index date only**				
OACs alone	25,910 (61.4)	767,156 (66.3)	1 [Reference]	1 [Reference]
SSRIs + OACs	804 (1.9)	16,571 (1.4)	1.41	1.31 (1.21-1.41)
**15 days before index date**				
OACs alone	29,652 (70.3)	847,662 (73.3)	1 [Reference]	1 [Reference]
SSRIs + OACs	1,003 (2.4)	19,954 (1.7)	1.41	1.30 (1.21-1.39)

CI = confidence interval, OAC = oral anticoagulant, IRR = incidence rate ratio, SSRI = selective serotonin reuptake inhibitor

a. Use of SSRIs alone, non-SSRI antidepressants alone, multiple users, and non-users were also included in the model for proper estimation of treatment effect.

b. Cases and controls were matched for age, sex, calendar year of cohort entry, and duration of follow-up.

c. IRR after matching of cases and controls.

d. Adjusted for all variables listed in Table 1.

eTable 8. Crude and Adjusted IRRs of Major Bleeding Associated With Concomitant Use of SSRIs With OACs, With Covariates Measured Prior to Cohort Entry.[a]

	No. (%) of Participants			
	**Cases (n=42,190)**	**Controls[b] (n=1,156,641)**	**Crude IRR[c]**	**Adjusted IRR[d] (95% CI)**
OACs alone	31,417 (74.4)	881,988 (76.3)	1 [Reference]	1 [Reference]
SSRIs + OACs	1,127 (2.7)	21,708 (1.9)	1.43	1.32 (1.24-1.42)

CI = confidence interval, IRR = incidence rate ratio, OAC = oral anticoagulant, SSRI = selective serotonin reuptake inhibitor
a. Use of SSRIs alone, non-SSRI antidepressants alone, multiple users, and non-users were also included in the model for proper estimation of treatment effect.
b. Cases and controls were matched for age, sex, calendar year of cohort entry, and duration of follow-up.
c. IRR after matching of cases and controls.
d. Adjusted for all variables listed in Table 1.

© 2024 Rahman AA et al. *JAMA Network Open.*

eTable 9. Crude and Adjusted IRRs of Major Bleeding Associated With Concomitant Use of SSRIs With OACs, After Multiple Imputation of Missing BMI and Smoking Values.[a]

	No. (%) of Participants			
	**Cases (n=42,190)**	**Controls[b] (n=1,156,641)**	**Crude IRR[c]**	**Adjusted IRR[d] (95% CI)**
OACs alone	31,417 (74.4)	881,988 (76.3)	1 [Reference]	1 [Reference]
SSRIs + OACs	1,127 (2.7)	21,708 (1.9)	1.43	1.31 (1.23-1.39)

CI = confidence interval, IRR = incidence rate ratio, OAC = oral anticoagulant, SSRI = selective serotonin reuptake inhibitor
a. Use of SSRIs alone, non-SSRI antidepressants alone, multiple users, and non-users were also included in the model for proper estimation of treatment effect.
b. Cases and controls were matched for age, sex, calendar year of cohort entry, and duration of follow-up.
c. IRR after matching of cases and controls.
d. Adjusted for all variables listed in Table 1.

© 2024 Rahman AA et al. *JAMA Network Open.*

eTable 10. Crude and Adjusted IRRs of Major Bleeding Associated With Concomitant Use of SSRIs With OACs, Compared With OAC Use Alone, by Type of OAC and Excluding Patients With Valvular AF.

	**No. (%) of Participants**			
	**Cases (n=41,313)**	**Controls[a] (n=1,113,921)**	**Crude IRR[b]**	**Adjusted IRR[c] (95% CI)**
**DOACs[d]**				
DOACs alone	9,396 (22.7)	265,828 (23.9)	1 [Reference]	1 [Reference]
SSRIs + DOACs	343 (0.8)	6,980 (0.6)	1.42	1.26 (1.12-1.41)
**VKAs[e]**				
VKAs alone	21,379 (51.8)	585,125 (52.5)	1 [Reference]	1 [Reference]
SSRIs + VKAs	757 (1.8)	13,864 (1.2)	1.46	1.35 (1.25-1.48)

CI = confidence interval, IRR = incidence rate ratio, OAC = oral anticoagulant, SSRI = selective serotonin reuptake inhibitor
a. Cases and controls were matched for age, sex, calendar year of cohort entry, and duration of follow-up.
b. IRR after matching of cases and controls.
c. Adjusted for all variables listed in Table 1.
d. Use of VKAs alone, SSRI + VKAs, SSRIs alone, non-SSRI antidepressants alone, multiple users, and non-users were also included in the model for proper estimation of treatment effect.
e. Use of DOACs alone, SSRI + DOACs, SSRIs alone, non-SSRI antidepressants alone, multiple users, and non-users were also included in the model for proper estimation of treatment effect.

© 2024 Rahman AA et al. *JAMA Network Open.*

eTable 11. Adjusted HRs of Major Bleeding Associated With Concomitant Use of SSRIs With OACs Compared With OAC Use Alone, in a Time-Conditional Propensity Score-Matched Analysis.[a]

Exposure	Number of patients	Events	Person Years	Incidence rate[b] (95% CI)	Adjusted HR[c] (95% CI)
OACs alone	12,192	613	21,797	28.1 (25.9-30.4)	1 [Reference]
SSRIs + OACs	12,192	373	9,902	37.7 (33.9-41.7)	1.23 (1.08-1.40)

CI = confidence interval; HR = hazard ratio; OAC = oral anticoagulants.
a. Propensity score computed from logistic regression model conditioned on all variables listed in Table 1
b. Incidence rates are expressed per 1,000 persons per year.
c. Matched on age, sex, calendar date of cohort entry of OAC initiation, and time-conditional propensity score.

© 2024 Rahman AA et al. *JAMA Network Open.*

eTable 12. Crude and Adjusted IRRs of Major Bleeding Associated With Concomitant Use of SSRIs With OACs, With Adjustment for Additional Comedications Interacting With OACs.[a]

	No. (%) of Participants			
	Cases (n=42,190)	Controls[b] (n=1,156,641)	Crude IRR[c]	Adjusted IRR[d] (95% CI)
OACs alone	31,417 (74.4)	881,988 (76.3)	1 [Reference]	1 [Reference]
SSRIs + OACs	1,127 (2.7)	21,708 (1.9)	1.43	1.31 (1.23-1.39)

CI = confidence interval, IRR = incidence rate ratio, OAC = oral anticoagulant, SSRI = selective serotonin reuptake inhibitor
a. Use of SSRIs alone, non-SSRI antidepressants alone, multiple users, and non-users were also included in the model for proper estimation of treatment effect.
b. Cases and controls were matched for age, sex, calendar year of cohort entry, and duration of follow-up.
c. IRR after matching of cases and controls.
d. Adjusted for all variables listed in Table 1 and the following comedications: clarithromycin, erythromycin, penicillin, azole antifungals, quinidine, amiodarone, dronedarone, propafenone, allopurinol, oral corticosteroids, tamoxifen, valproic acid, cyclosporin, tacrolimus, disulfiram, methylphenidate, and sulfamethoxazole.

© 2024 Rahman AA et al. *JAMA Network Open.*

eReferences.

1. VanderWeele, T.J. and M.J. Knol, *A Tutorial on Interaction.* Epidemiologic Methods, 2014. **3**(1): p. 33-72.
2. Rothman, K.J., *Epidemiology: an introduction, 2nd edition*. 2012, New York: Oxford University Press, Inc.
3. Suissa, S., E.E. Moodie, and S. Dell'Aniello, *Prevalent new-user cohort designs for comparative drug effect studies by time-conditional propensity scores.* Pharmacoepidemiol Drug Saf, 2017. **26**(4): p. 459-468.
4. Suissa, S., S. Dell'Aniello, and C. Renoux, *The prevalent new-user design for studies with no active comparator: The example of statins and cancer.* Epidemiology, 2023: p. 681-689.

© 2024 Rahman AA et al. *JAMA Network Open.*
