# Supplementary material for: Concomitant Use of Selective Serotonin Reuptake Inhibitors With Oral Anticoagulants and Risk of Major Bleeding
Source: JAMA Netw Open. 2024 Mar 22;7(3):e243208. doi: 10.1001/jamanetworkopen.2024.3208 (PMC10960200; doi:10.1001/jamanetworkopen.2024.3208)
Supplement: Supplement 2. — Data Sharing Statement [file jamanetwopen-e243208-s002.pdf]

Data Sharing Statement

Rahman. Concomitant Use of Selective Serotonin Reuptake Inhibitors With Oral Anticoagulants and Risk of Major Bleeding. *JAMA Netw Open*. Published March 22, 2024. doi:10.1001/jamanetworkopen.2024.3208

Data

Data available: This study is based in part on data from the Clinical Practice Research Datalink obtained under license from the UK Medicines and Healthcare products Regulatory Agency. The data are provided by patients and collected by the UK National Health Service as part of their care and support. The interpretation and conclusions contained in this study are those of the author/s alone. Because electronic health records are classified as "sensitive data" by the UK Data Protection Act, information governance restrictions (to protect patient confidentiality) prevent data sharing via public deposition. Data are available through the individual constituent entities controlling access to the data. Specifically, the primary care data can be requested via application to the Clinical Practice Research Datalink (https://www.cprd.com).
